# Effects of acute exercise on directed forgetting

**DOI:** 10.34172/hpp.2020.61

**Published:** 2020-11-07

**Authors:** Paul D. Loprinzi, Jacob Harper, Tatjana Olinyk, Jessica Richards

**Affiliations:** Exercise & Memory Laboratory, Department of Health, Exercise Science and Recreation Management, The University of Mississippi, University, MS 38677, USA

**Keywords:** Cognition, Exercise, Physical activity

## Abstract

**Background:** The directed forgetting paradigm involves individuals encoding a list of words(List 1; L1) and then, prior to encoding a second list of words (List 2; L2), they are given specific instructions to either remember all the words from L1 or to try and forget these words. In this paradigm, after encoding L1, those who are given the directed forgetting (DF) instructions tend tore call more words for L2 when compared to those who were given the remember (R) instructions(DF benefit effect). Similarly, those given the DF instructions tend to recall fewer words from L1(DF cost effect). This DF phenomenon may, in part, occur via attentional inhibitory mechanisms, or mental context-change mechanisms, which may be influenced via acute exercise.

**Methods:** The present experiment investigates if acute exercise can facilitate DF when exercise occurs after L1 forgetting instructions. Participants (N = 97; M_age_ = 21 years) were randomly assigned into either acute exercise (15-min high-intensity aerobic exercise) plus DF (EX + DF),2) DF (directed forgetting) only (DF) or 3) R (remember) only (R). A standard two list (L1 and L2)DF paradigm was employed.

**Results:** We observed evidence of a DF cost effect, but not a DF benefit effect. For L1, although both EX + DF and DF differed from R, there was no difference between EX + DF and DF. Further, although for L2, EX + DF was different than DF, neither of these groups differed when compared to R.

**Conclusion:** We reserve caution in suggesting that exercise had a DF effect.

## Introduction

In the common directed forgetting paradigm, individuals encode an array of words (List 1) and then are given specific instructions to either remember or forget the List 1 words. Intentional forgetting of List 1 may help to more successfully encode and recall a subsequent list of words (List 2). Thus, in this standard directed forgetting paradigm, after encoding List 1, those who are given the directed forgetting (DF) instructions tend to recall more List 2 words when compared to those who were given the remember (R) instructions after List 1.^1^ This phenomenon is called the *benefits* of DF. When the forget instructions reduces recall of List 1 items relative to the R group, this is referred to as the *costs* of DF.

Previous research demonstrates that acute exercise, including high-intensity continuous exercise,^2,3^ may increase the ability to recall lists of words.^4^ Whether this effect occurs through mechanisms related to encoding, consolidation, or retrieval is not clear. However, recent work indicates that acute exercise may facilitate memory through each of these memory phases.^5^

Although accumulating research has investigated the influence of acute exercise on single memory performance, what is lacking in the literature is whether acute exercise influences forgetting. In addition to enhancing the ability to recall lists of words, emerging research has investigated the influence of acute exercise on directed forgetting of word lists. In our previous study, which was the first experiment on this topic, we evaluated whether acute exercise could facilitate *selective* forgetting of List 1 words (e.g., only List 1 words printed in a specific color).^6^ We failed to demonstrate any exercise-related effect. As a follow-up to our first experiment, in our second experiment, instead of focusing on selective forgetting, we specifically evaluated whether acute exercise could augment the beneficial effects of direct forgetting on List 2.^7^ In these two experiments, we demonstrated a directed forgetting benefit (List 2 enhancement effect) but failed to demonstrate any ability for acute exercise to augment this directed forgetting effect.

In our past two experimental studies on this topic, acute exercise occurred shortly before encoding List 1. In the present experiment, we positioned the acute bout of exercise between List 1 and List 2. Cognitive inhibition, in part, may be responsible for the directed forgetting effect.^8^ That is, after being told to forget List 1, the prefrontal cortex may engage in inhibitory processes to suppress the learned material (List 1). For example, from a retrieval inhibitory account, inhibitory processes may prevent the retrieval of “to-be-forgotten” items at testing. The specific “forget” instructions may initiate a process that serves to inhibit access routes to the target items that were previously encoded. We specifically theorized that engaging in acute exercise at this point (after L1 instructions) would facilitate this effect, given that other research has shown that exercise can influence cognitive inhibition.^9^ Thus, for the present experiment, we hypothesized that, after encoding List 1, those who exercised and received DF instructions would have a greater List 2 recall (DF benefits) and a lower List 1 recall (DF costs) when compared to those only receiving DF instructions.

## Material and Methods

### Study design

All participants consented to participate in this study. Participants were randomly assigned into either 1) exercise plus DF (EX + DF), 2) DF (directed forgetting) only (DF) or 3) R (remember) only (R). The protocol for these groups is shown in Table 1.

### Participants

The total sample included 97 participants, which is considerably higher than other related research on this subject matter (N = 60).^7^ This was based on an a-priori power analysis indicating that at least 93 participants would be needed for the following inputs, α = 0.05, power = 0.85, 3 groups, 2 within-subject measurements, and a partial η2 of 0.03. Participants (18-26 years), including undergraduate and graduate students, were recruited via convenience sampling. Additionally, participants were excluded if they were a smoker, pregnant, exercised five hours before the visit, consumed caffeine three hours before the visit, had a head trauma in the past month, consumed mind-altering substances in the past month or were a daily alcohol user.

### Exercise protocol

The exercise arm (Ex + DF) exercised, on a treadmill, at 80% of their heart rate reserve (HRR), constituting high-intensity exercise.^10^ High-intensity exercise was specifically chosen as emerging research demonstrates that high-intensity exercise may be more effective in improving memory and executive function when compared to lower-intensity acute exercise.^11^ During the bout of exercise, the speed and incline were manipulated to maintain a heart rate close to 80% of the participant’s HRR. As shown in Table 1, this bout of exercise occurred after List 1.

### Non-exercise protocol

The DF and R groups completed a time-matched 20-minute seated task. This involved watching (self-selected) either The Office or Big Bang Theory. Previous research suggests that this is a suitable control task.^12^

### Memory assessment

The directed forgetting paradigm employed for this experiment was identical to our past research.^7^ In brief, this involved two lists (List 1 and List 2; L1 and L2), with each list comprised of 16 unrelated words from the Toronto Word Pool. Words were presented in a set random order, displayed individually at a rate of 4-seconds followed by a 1-second interstimulus interval. The outcome variable was the number of words recalled from L1 and L2.

After L1 presentation, and depending on group assignment, participants received specific instructions. As detailed elsewhere,^7^ those in the DF group were told to forget the words from L1, whereas those in the R group were told to remember all the L1 words.

After L2, for 30-seconds, participants completed a distractor task (math problems). Following this distractor task, free recall was performed verbally. The order of L1 and L2 were counterbalanced at both encoding and recall.

### Statistical analysis

A 2 (L1, L2) x 3 (Ex + DF, DF, R) analysis of variance was computed, with no violations of its respective analytical assumptions. The within-subject factor included two levels (L1 and L2), whereas the between-subject factor included three levels (Ex + DF, DF, R). An alpha of 0.05 was used to denote statistical significance, with eta-squared (η^2^) values calculated as a measure of effect size. All analyses were computed in JASP (v. 0.13.1.0; The Netherlands) and there were no missing data.

## Results

Table 2 shows the sample characteristics. Table 3 shows the physiological (HR) data for the three conditions.

**Figure 1:**
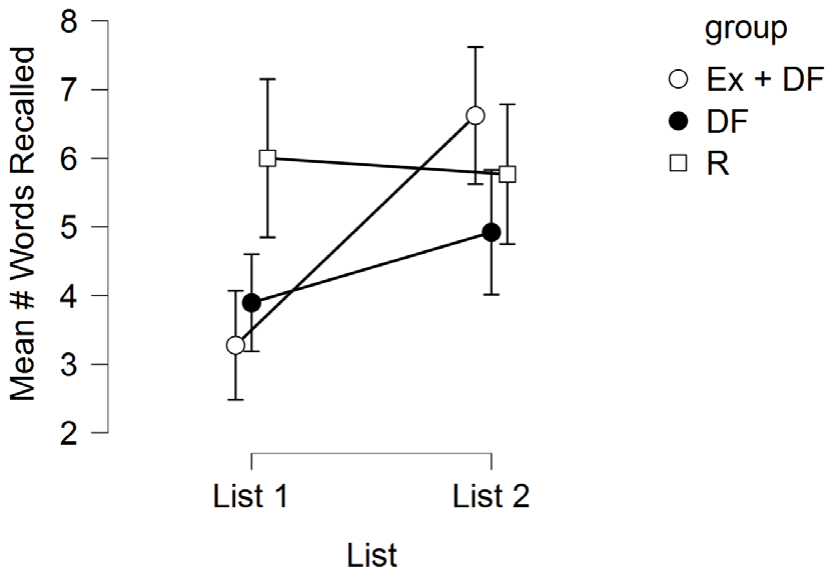
Mean number of words recalled across the experimental conditions. Error bars are 95% CI

The memory scores for the three experimental conditions are displayed in Figure 1. There was a statistically significant main effect for list, *F* (1, 94) = 13.82, *P* < 0.001, η^2^ = 0.06, main effect for group, *F* (2, 94) = 5.38, *P* = 0.006, η^2^ = 0.09, and a significant list by group interaction, *F* (2, 94) = 7.39, *P* = 0.001, η^2^ = 0.06. Notably, results were similar when controlling for various demographic parameters (age, gender, race-ethnicity, BMI).

Post-hoc tests demonstrated evidence of a DF *cost* effect. That is, for L1, EX + DF was lower than R (*P* = 0.0002), and similarly, DF was lower than R (*P* = 0.001). However, for L1, EX + DF was not different than DF (*P* = 0.24). Although for L2, EX + DF was greater than DF (*P* = 0.01), we did not observe evidence of a DF *benefit* effect. That is, for L2, EX + DF was not different than R (*P* = 0.22), and similarly, DF was not different than R (*P* = 0.21).

## Discussion

The present experiment was designed as a follow-up of previous experimental work on this topic.^6,7^ The main finding of the present experiment was that we observed evidence of a DF cost effect, but not a DF benefit effect. For L1, although both EX + DF and DF differed from R, there was no difference between EX + DF and DF. Further, although for L2, EX + DF was different than DF, neither of these groups differed when compared to R. As such, we reserve caution in suggesting that exercise had a DF effect.

As we have discussed elsewhere,^7^ various theoretical accounts for DF have been suggested, which include selective rehearsal, active erasure, and tagging and selective search. Within the context of exercise, the inhibitory account of DF may be a contributor to any potential exercise-related effect on DF. The attention inhibition theory posits that an active process is in place to mitigate the accessibility of the forgotten items. After L1, when given the directed forgetting instruction, L1 items may be suppressed via an effortful inhibitory process. In our past two experiments, we placed the acute bout of exercise prior to L1 encoding, which may have been a less than ideal temporal positioning. As we did in the present experiment, we thought that placing the acute bout of exercise occurring immediately after the DF instructions would help to prime attentional inhibitory mechanisms. Past work, indeed, has demonstrated that both chronic exercise^13^ and acute exercise^14^ can improve attentional control in young adults. Recent neuroelectrical work suggests that exercise may facilitate attentional inhibition via alterations in N450 and P3 neural markers.^14^

As discussed elsewhere,^15^ inhibitory mechanisms in the attentional network are not a unitary phenomenon. The orienting network (frontal lobe, posterior parietal lobe, midbrain and thalamus) participates in locating relevant objects in space and filtering out irrelevant information that could influence attention. The executive network (frontal lobe) is instrumental in self-regulation and situations that involve control-oriented processes. These networks, in isolation, may help to avoid re-examinations, either by preventing reiterative attention to previous explored locations (orienting network) or by preventing attention from returning to a non-spatial component of the stimuli (executive network).^15^ In theory, acute exercise may help prime this executive network, and thus, prevent attention from returning to rehearsing or re-experiencing the L1 stimuli. In support of this, recent fMRI work has shown that acute exercise increased synchrony among brain regions involved in attention and executive control.^16^

However, despite this plausibility through which exercise may help to facilitate DF, the present results, along with our previous experiments, does not provide convincing evidence that acute exercise can influence DF costs or benefits. In addition to the novelty of evaluating exercise on this paradigm (DF), another notable aspect of this study was integrating a delay period within the DF framework. Very few studies have examined DF after a delay.^17^

A limitation of this experiment is the unequal distribution of gender across the conditions. However, we computed additional sensitivity analyses that controlled for gender and our RM-ANOVA results were similiar (results not shown). Another limitation is that we did not evaluate the participant’s cardiorespiratory fitness, which may be useful to consider in future work, as, in theory, fitness may moderate the effects of high-intensity exercise on cognition.

## Conclusion

In conclusion, in our two previous experiments, acute exercise did not augment the effects of directed forgetting when the bout of exercise occurred prior to the forgetting instructions. In the present experiment, we also did not observe consistent evidence of an effect of exercise on DF. Future work should continue to investigate this understudied topic. It is possible that the delay period used in the present experiment made it difficult to observe a DF benefit, and thus, an exercise-induced DF effect. To avoid this potential issue, research should re-evaluate the influence of exercise on DF when the bout of exercise is placed prior to L1. For such work, and as addressed thoroughly elsewhere,^17^ studies should consider implementing a four-list DF paradigm, where the forget/remember cue is varied within-subjects. Including this remember control group via the four-list design may provide a greater systematic assessment of the costs and benefits of DF. Lastly, future work should consider that, if exercise does have a DF effect, perhaps it acts through non-inhibitory mechanisms. For example, as discussed elsewhere,^17^ DF may occur from a context-change in mental state. Individuals with greater working memory capacity may have a greater ability for context encoding, and ultimately, show a larger effect for the forget cue. Exercise may benefit DF here by enhancing working memory capacity.^18^ Clearly, future research on this under-investigated topic is needed. This research should determine whether acute exercise does indeed influence DF, and if so, what the prevailing mechanism(s) is.

## Funding

This study did not involve any funding.

## Competing interests

All authors have no competing interests.

## Ethical approval

The ethics committee at the authors’ institution approved this study (#19-041).

## Authors’ contributions

Author PL conceptualized the study, supervised the experiment and developed the first draft of the manuscript. All authors approved the final manuscript version and assisted in data collection.

**Table 1:** Study protocol

**Group**	**Start – – – – – – – – – – – – – – – – – – – – – – – – – – – – – – – – – – – – – – – – – – – – – – – – – – – – – – – – – – – – – – – – – – – – – – – – – – – – →**						
EX + DF	Forms/surveys	List 1 encoding	Instructions to forget	15-min exercise, 5-min video	List 2 encoding	30-seconds of math	Memory recall
DF	Forms/surveys	List 1 encoding	Instructions to forget	20-min video	List 2 encoding	30-seconds of math	Memory recall
R	Forms/surveys	List 1 encoding	Instructions to remember	20-min video	List 2 encoding	30-seconds of math	Memory recall

**Table 2:** Characteristics of the sample

**Variable**	**Ex + DF**	**DF**	**R**
N	29	38	30
Age, mean years	21.0 (1.5)	21.4 (2.7)	20.9 (1.7)
Gender, % Female	37.9	68.4	66.6
Race-Ethnicity, % White	69.0	100	100
BMI, mean kg/m^2^	25.5 (3.7)	25.0 (6.5)	25.5 (4.1)

BMI, body mass index. Values in parentheses are standard deviations

**Table 3:** Heart rate responses

**Variable**	**Ex + DF**	**DF**	**R**
Rest, mean bpm	74.2 (7.7)	75.3 (10.3)	78.9 (10.4)
Midpoint of Exercise, mean bpm	168.1 (11.6)	-	-
Endpoint of Exercise, mean bpm	169.7 (15.6)	-	-
5-min Post Exercise, mean bpm	99.2 (11.5)	-	-

Values in parentheses are standard deviations. Dashed line (-) indicates that the measurement was not taken.
